# A longitudinal observation study assessing changes in indicators of serious injury and violence with alcohol controls in four remote indigenous Australian communities in far north Queensland (2000–2015)

**DOI:** 10.1186/s12889-018-6033-1

**Published:** 2018-09-17

**Authors:** Alan R. Clough, Michelle S. Fitts, Reinhold Muller, Valmae Ypinazar, Stephen Margolis

**Affiliations:** 1 Community-based Health Promotion and Prevention Studies Group, College of Public Health, Medicine and Veterinary Sciences, Australian Institute of Tropical Health and Medicine, James Cook University, PO Box 6811, Cairns, QLD 4870 Australia; 2 College of Public Health, Medicine and Veterinary Sciences, James Cook University, 1 James Cook Drive, Townsville, QLD 4811 Australia; 3 Griffith University, Mt Gravatt, Brisbane, QLD 4122 Australia

**Keywords:** Alcohol, Alcohol supply controls, Indigenous Australia, Injury, Violence

## Abstract

**Background:**

Legal restrictions on alcohol availability have been used to address violence and injury in the world’s remote Indigenous communities. In Australia, alcohol management plans (AMPs) were implemented by the Queensland Government in 2002. This study reports changes in indicators of alcohol-related violence and injury in selected communities.

**Methods:**

*Design and setting:* A longitudinal observational study was conducted in four Aboriginal and Torres Strait Islander (Indigenous) communities in Cape York, far north Queensland. All communities are similarly-isolated from population centres where alcohol is available.

*Data:* For 2000 to 2015 inclusive: 1019 Royal Flying Doctor Service aeromedical trauma retrievals; 5641 Queensland Police Service records of unique assault occurrences, including 2936 involving alcohol; and records for 2741 unique assault victims were examined.

*Data analysis:*

Rates (per 1000 population) of trauma retrievals, assault occurrences and assault victims (per 1000 population) were compared across three policy phases.

Phase 1: 2000 to 2008. Initial restrictions on possession and consumption of alcohol in ‘restricted areas’ were implemented during 2002–2003.

Phase 2: 2009 to 2012. All alcohol was prohibited in three study communities and its legal availability limited in the fourth from 2009.

Phase 3: 2013 to 2015. Government reviews of AMP policies in light of legal challenges and community responses characterise this phase.

**Results:**

Compared with Phase 1, in Phase 2 retrieval rates declined by − 29.4%, assault occurrences by − 34.1% with less than one-third involving alcohol, and assault victims by − 21.1%, reaching historically low levels in 2010–2012. These reductions did not continue consistently. Compared with Phase 1, in Phase 3 retrieval rates, assault occurrence rates and assault victim rates declined by somewhat lesser amounts, − 13.9%, − 15.0% and − 13.4%, respectively. In Phase 3, the proportion of assault occurrences involving alcohol in communities 2, 3 and 4 rose towards pre-2008 levels.

**Conclusions:**

Early successes of these controversial alcohol restrictions are jeopardised. Indicators of violence and injury appear to be rising once more in some AMP communities. Importantly, rates have not generally exceeded the highest levels seen in Phase 1. Fresh policy action is required with rigorous monitoring to prevent erosion of initial important successes.

## Background

With 5.1% of the global burden of disease and injury attributable to alcohol [1], legal restrictions on alcohol’s physical availability in the general population are key among alcohol control strategies used by governments in most developed economies [2]. For the Indigenous populations in developed economies such as Canada [3], the United States [4] and Australia [5], legal restrictions on alcohol have been specifically designed and used targeting populations in remote localities. Where rigorous evaluations are available [6–10], such targeted interventions have generally shown favourable effects, at least initially [11].

In Australia, legal restrictions on alcohol’s physical availability in Indigenous communities became known as ‘Alcohol Management Plans’ (AMPs) towards the end of the twentieth century [12–14]. AMPs have now become part of the national policy infrastructure aimed at reducing the burden of disease and injury for Aboriginal and Torres Strait Islander (Indigenous) Australians [15]. In Queensland, AMPs are currently in place in 19 Indigenous communities (situated within 15 Local Government Areas); singled out in a Government-commissioned inquiry (published in 2001) as among the State’s most vulnerable [12, 13, 16]. Queensland’s AMPs involve ‘restricted area’ declarations under S173G (Part 6A) of the *Liquor Act 1992* with controls on quantities and types of alcohol permitted in a restricted area designated under S173H [17] and set out in Schedules 1A-1R of the *Liquor Regulation 2002* [18].

As already described in detail in a previous publication, these place-based controls were first introduced in 2002 [19]. By 2006, AMPs had been implemented in all 19 communities [19]. From 2007, AMPs were reinforced with ‘harm minimisation’ measures to control the supply of alcohol from licensed premises located near the AMP communities targeted to receive the intervention [19]. In 2008, further legislative measures aimed at closing community ‘canteens’ and ‘taverns’ [20, 21] tightened access to alcohol in *all* of the 19 AMP communities; bringing complete prohibition to seven of them [19].

At the end of 2012, the Queensland Government implemented formal processes to review AMPs [5, 19]. As this phase of review has unfolded, systematic evidence from key service providers and community leaders in affected communities and neighbouring rural centres has suggested that initial significant reductions in violence and improved community amenity and safety, were comparatively short-lived, with many localities seeing unintended impacts linked with continued access to illicit alcohol [22–25], a factor which has consistently undermined the effectiveness of liquor restrictions in rural and remote Australia [14, 22, 26] and in similar settings elsewhere [8, 27, 28]. The ongoing effectiveness of Queensland’s AMPs therefore requires robust assessment and monitoring.

Using data provided by Australia’s Royal Flying Doctor Service (RFDS), we have monitored rates of aeromedical retrievals for serious injury in four AMP communities over the 15 year period from 1995 to 2010 [7, 10]. This is already the longest period of monitoring for any indicator in Australian evaluations [9, 14, 29, 30]. Significant reductions in this important surrogate measure of alcohol-related violence and injury were documented in the selected sentinel communities after the first round of AMP restrictions in 2002–03 but rising during the following two years [7] before falling after the second round of restrictions in 2008 to historically low levels by 2010 [10]. In this study the monitoring period is extended into the current important phase of AMP review, from the end of 2012, offering a methodologically strengthened evaluation. The study updates the RFDS information about numbers of aero-medical retrievals for serious injury for the same four communities and combines it with additional independent surrogate measures of alcohol-related violence, namely rates of occurrences of offences and victims of all ‘person-to-person’ violence recorded by the Queensland Police Service (QPS). Data for the three time series was available for the period 2000 to 2015 (inclusive). The study specifically examines the issue of whether the important successes achieved after 2008 have been sustained beyond 2010 into the current review phase. The policy environment characterising the current review phase is briefly described and the patterns and trends seen in the injury and violence indicators are interpreted in this light. The implications for future policy action and monitoring are considered.

## Methods

### Setting

The communities, their populations and the reasons for choosing them as sentinel communities for monitoring have already been described in detail [7, 10]. In summary, the communities are: of similar size; located in the same remote region of north Queensland and are all similarly isolated from significant population centres where alcohol is available. The RFDS remains the principal service transporting patients to acute care at the region’s tertiary hospitals, and QPS has an enduring mandate to enforce Queensland’s *Criminal Code 1995* with a local police presence in most remote communities, including the four in this study. This analytical approach ensures that the indicators examined reflect changing circumstances in these four communities, independent as far as practicable from external confounding factors, providing the opportunity to consider the local impacts of Queensland-wide policy initiatives and shifts.

### Phases of alcohol restrictions and review

This longitudinal observational study assesses the effects of a policy intervention which was developed and implemented with a changing focus over the past two decades. The very complex legislative and regulatory background for AMPs in all Queensland Indigenous communities has already been described and summarised [19]. The four study communities, as with all communities affected by AMPs, were subjected to a stepwise tightening of restrictions through legislation and regulation which, from 2002, progressively reduced alcohol’s legal availability [7, 10, 19]. Three phases of alcohol controls, since the beginning of the study period in 2000, can be outlined.

#### Phase 1 – From 2000 to 2008: The first round of restrictions

Since the 1980s, each community’s elected Local Government Council (LGC) had been operating licensed premises, variously known as a ‘tavern’ or ‘canteen’ [17], from which beer was sold legally for consumption on-premises or to be taken away. This source of alcohol underpinned high levels of consumption [10, 19, 31, 32]. With no restrictions, consumers could also bring alcohol into the community, sourced legitimately from the accessible regional towns and centres, and this reinforced the established harmful consumption patterns [7, 13, 16, 32]. Although community groups had been empowered since the mid-1990s to declare “controlled” or “dry” places and to limit the possession of alcohol in some settings inside community area boundaries, there had been few substantial, robust controls on alcohol availability and consumption in the four communities for over a decade [19]. During the 1990s, public health researchers [32] and social justice [13] and Indigenous advocates [16, 33] consistently urged strenuous Queensland Government intervention.

Following Justice Fitzgerald’s 2000 inquiry [13], over a 12 month period from the end of 2002 to late in 2003, all four communities were declared ‘restricted areas’ and the quantities and types of alcohol permitted in each were prescribed [10, 19]. The initial effect was to prohibit the possession and consumption of higher alcohol content products in one study community while no alcohol could be possessed and consumed in the other three, beyond the licensed areas of their still-operating ‘tavern’ or ‘canteen’ [18].

During 2007, legislation and regulation to control alcohol availability across the wider region saw ‘minimising harm’ conditions placed on the licences of liquor retailers in the ‘catchment’ areas of restricted area communities [19]. Retailers were required to not sell other than the permitted quantities and types of alcohol to patrons they knew, or believed would transport the alcohol into a restricted area [19]. The transition year 2008 saw a raft of legislative amendments and initiatives implemented to make all AMP communities ‘as dry as possible’ [19, 21].

#### Phase 2 – From 2009 to 2012: ‘as dry as possible’

In 2008, the *Liquor Act 1992* was amended removing the option for LGCs to hold a liquor licence while also making it an offence to ‘attempt’ to bring prohibited quantities and types of liquor into a restricted area [19, 21]. For three of the study communities this meant that their local ‘canteen’ or ‘tavern’ was closed, thereby prohibiting all alcohol from 2009. In the fourth community, the ‘canteen’ was turned into a social club by early 2009, operating under a ‘Restricted Liquor Permit’ [17]. The on-premises purchase and consumption of liquor containing ≤4% alcohol by volume was permitted in this community, but the possession and consumption of any alcohol off-premises remained prohibited in the wider restricted community area [19].

#### Phase 3–2013 to 2015: The current review period

Significant legal challenges were mounted to the legitimacy of Queensland’s AMPs commencing during the previous Phase 2 [34, 35] and decided in this subsequent Phase 3 from 2013 [36–38]. In the lead up to Queensland’s March 2012 election, both major parliamentary parties proposed to review AMPs as part of their election platforms and a new Queensland Government formally announced the review in October 2012 [19]. Successive Queensland Governments since have sustained this policy position [25]. Additionally, during Phase 3, the Commonwealth Government has conducted reviews of the appropriateness of alcohol control measures in the Northern Territory, also called AMPs, implemented as part of its internationally controversial ‘Stronger Futures’ legislation addressing Indigenous disadvantage [39, 40].

While this important period of review and legal challenge has unfolded, liquor restrictions in the four communities have not changed since 2008, with one exception. In mid-2014, in one community with prohibition, a venue formerly run by the LGC was permitted to operate once again, with limited trading hours and also with the on-premises purchase and consumption of liquor containing ≤4% alcohol by volume permitted under a ‘Restricted Liquor Permit’ [17].

### Data

The causal mechanisms linking alcohol use and interpersonal violence are well known [1, 41, 42] with significant proportions of serious injuries linked with alcohol in the literature for the general population [43, 44]. For the communities in this study, it has been documented that 51% of injuries, including those requiring emergency aeromedical retrieval by the RFDS, were linked with alcohol during the 1990s [32]. Accordingly, this study updates the information already published about aeromedical retrievals for serious injury from the four communities.

In the general population, it is widely recognised that alcohol is involved in more than half of all occurrences of interpersonal violence with alcohol’s acute effects having the greatest impact on violent behaviour [45]. For the communities in this study, most alcohol-related injury was documented as due to assault during the 1990s [32]. We therefore used the indicator of occurrences of ‘violence against the person’ recorded by the QPS and the accompanying descriptor of any involvement of alcohol in each occurrence, where it was available.

In addition, since QPS records also include information about individuals affected by violent occurrences, and since the victims of assault often require some kind of acute care medical intervention, to capture as wide a spectrum of relevant violent incidents as possible, we used the indicator of victims of ‘violence against the person’ recorded by QPS.

### Aeromedical retrievals

A Government-funded clinic has provided local health care services in each study community throughout the observation period. The RFDS remains the principal provider of emergency medical transport to definitive care of all people presenting to the local clinic with serious injuries. There have been no substantive changes in RFDS aeromedical retrieval processes, logistics or management for these communities across the time frame of our previously published [7, 10] and current studies.

As in these previous studies [7, 10], serious injury is defined as injury requiring hospital treatment that is not manageable within the communities.

#### Occurrences of ‘violence against the person’ (assault)

The QPS has a presence in each of the four communities in the form of a Division station. Two of the stations, i.e. those in the two largest communities, are staffed by the equivalent of eight permanent officers and, in the smaller communities, by the equivalent of two permanent officers. Some LGCs employ Community Police Officers to assist. In 2008, to support the ‘dry as possible’ phase, the Queensland Government committed funding for additional officers across all 19 AMP communities [19]. Occasionally, to respond to critical incidents, additional QPS officers are sent to AMP communities, including those in this study, for brief periods. While QPS numbers in far north Queensland have grown steadily over the past decade, personal communication with QPS operations management indicates that there have been no significant additional permanent changes to QPS resources allocated to the study communities throughout the period of observation.

Data were provided by QPS for all occurrences of person-to-person violent offences using Australian Standard Offence Classification Group Codes for ordering offences [46] including ‘offences against the person’ (referred to as ‘assault’ for the purposes of this study). Data described occurrences of grievous assault (including homicide and manslaughter), serious assault, common assault and life endangering acts known generally to be linked with alcohol [41, 42, 47, 48]. Incidents due to other violent acts were included where the available evidence suggests specific links with alcohol in these communities including driving causing death, sexual assault, rape and stalking [13, 32, 49]. For each occurrence a description of the involvement of alcohol in the occurrence was provided, where information was available to police.

#### Victims of ‘violence against the person’ (assault)

For harm observed by police in connection with an assault occurrence, or experienced or reported by victims, data for the same types of person-to-person violent offences [46] were used. A unique victim identifier was provided by QPS to the researchers so that a victim count could be determined for the whole period and for each year of observation.

### Data analysis

Rates of RFDS trauma retrievals and QPS registered occurrences of assault and victims of assault (per 1000 population) were calculated for all years between 2000 and 2015 for all four communities (Table 1). Yearly population estimates for the four communities were obtained from the Queensland Government Statistician’s Office [50] which makes yearly estimates of populations for Queensland localities based on Australian Bureau of Statistics data compiled every five years [51]. The communities had an estimated total population of 3780 in 2015 [50]. Average rates over all communities were calculated for each year and for each of the three AMP phases. In addition, the proportion of assault occurrences involving alcohol were calculated for each year. To assess the communities’ overall response to alcohol restrictions, the percentage change in the average rates of serious injury retrievals, assault occurrences and assault victims for each of Phases 2 and 3 were compared with rates in Phase 1, i.e. before the ‘canteens’ or ‘taverns’ were closed (or modified in the case of one community) and before all alcohol was prohibited (neither carriage nor consumption in the restricted areas in all four communities).

**Table 1:** Rates (per 1000 population) of trauma, occurrences and victims of violence (assault)

		Phase 1									Phase 2				Phase 3		
		2000	2001	2002	2003	2004	2005	2006	2007	2008	2009	2010	2011	2012	2013	2014	2015
RFDS retrievals for trauma																	
Community	1	16.8	24.2	20.7	15.5	15.3	27.3	24.8	30.1	19.2	16.0	16.1	10.7	16.0	11.1	21.0	20.4
	2	14.0	23.0	15.8	13.8	8.7	13.4	15.1	10.2	21.3	13.8	6.3	13.5	19.5	20.2	17.7	27.1
	3	9.4	15.7	32.1	28.0	15.2	15.4	43.3	20.9	37.8	19.9	16.3	11.5	5.3	27.4	12.0	15.8
	4	17.8	12.9	32.2	22.5	27.3	24.1	12.9	17.0	22.7	23.8	18.8	12.6	11.0	13.6	5.3	19.6
	Average	14.5	19.0	25.2	19.9	16.6	20.0	24.0	19.6	25.2	18.3	14.4	12.1	12.9	18.1	14.0	20.7
Occurrences of assault recorded by QPS																	
Community	1	145.3	104.3	74.8	84.5	176.6	206.7	123.0	112.5	82.3	77.6	70.3	84.4	65.6	103.0	84.0	168.7
	2	215.2	225.2	151.2	121.1	92.2	82.1	119.5	97.7	75.1	83.6	58.0	56.7	81.6	107.5	148.8	151.5
	3	28.2	23.5	48.2	89.0	71.1	59.9	136.7	148.3	106.3	86.6	94.2	82.7	40.3	75.8	22.4	35.9
	4	53.4	95.5	107.7	127.0	128.4	107.5	156.2	88.1	57.4	57.9	75.3	67.1	67.1	48.8	68.2	96.7
	Average	110.5	112.1	95.5	105.4	117.1	114.1	133.8	111.7	80.3	76.4	74.5	72.7	63.7	83.8	80.8	113.2
Victims of assault recorded by QPS																	
Community	1	87.5	75.4	53.2	43.6	99.1	105.6	79.0	67.0	48.7	42.6	59.3	60.8	56.9	61.5	59.3	130.9
	2	119.1	127.1	89.9	95.5	65.0	63.9	71.5	65.1	62.1	68.9	50.8	47.7	52.9	73.9	85.7	91.3
	3	15.7	14.1	49.8	70.8	47.4	41.1	74.4	89.0	61.3	72.2	68.8	71.2	28.0	53.2	20.9	21.6
	4	42.1	51.8	86.8	86.8	112.4	69.0	80.5	61.8	49.8	44.6	60.8	53.1	46.6	31.2	41.4	57.5
	Average	66.1	67.1	69.9	74.2	81.0	69.9	76.3	70.7	55.5	57.1	59.9	58.2	46.1	55.0	51.8	75.3

**Fig. 1:**
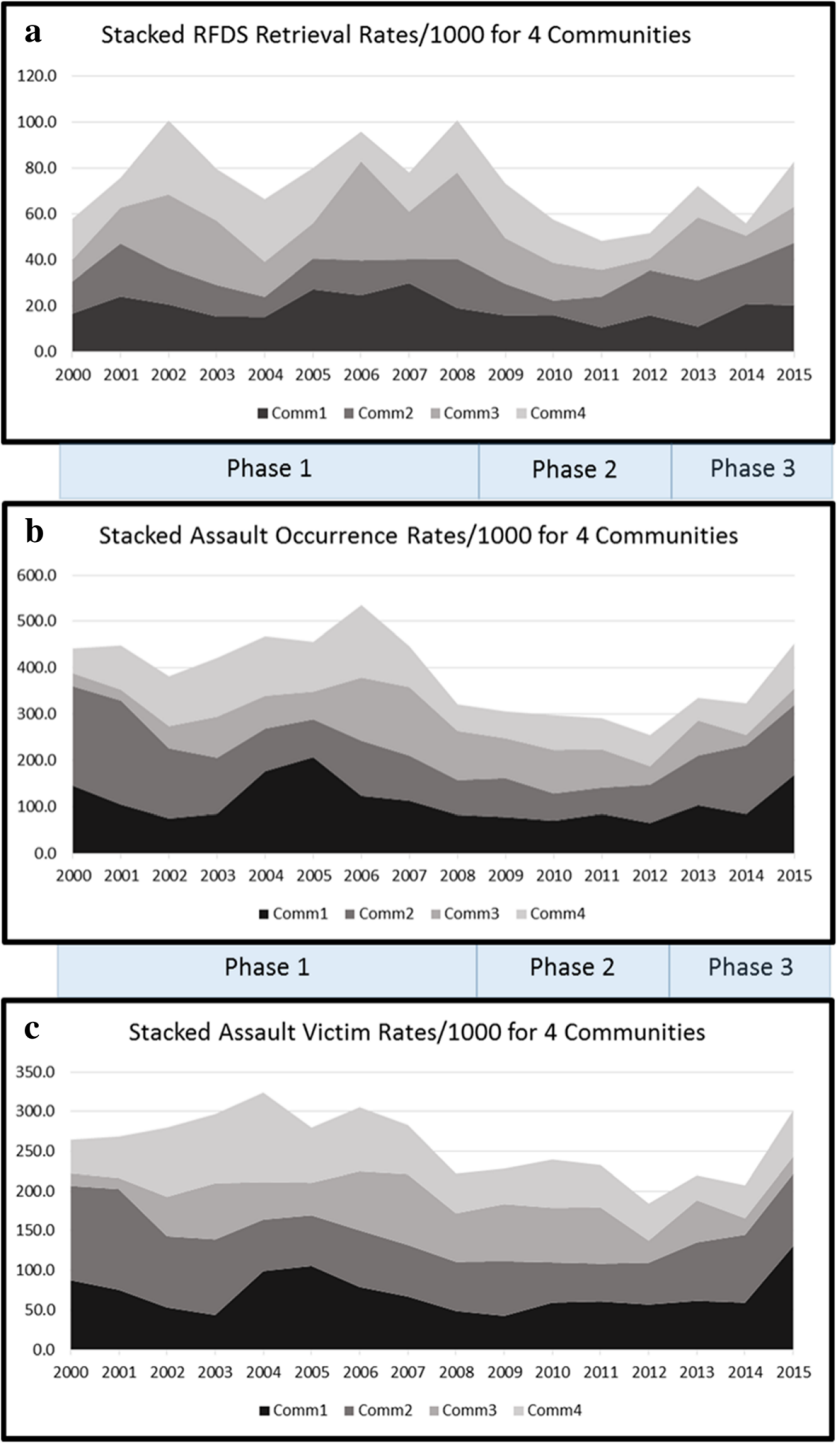
Rates (per 1000 population) for (**a**) trauma, (**b**) assault occurrences and (**c**) assault victims

Stacked “mountain graphs” were produced for retrieval rates (Fig. 1a), assault occurrence rates (Fig. 1b) and assault victims rates (Fig. 1c) over the study period. This type of graph allows the display of detailed community-specific time trends over the observation period. While community-specific rates can be directly gathered from the graphs in Fig. 1, the overall rate for the four communities is graphically inflated in these depictions by a factor of four (stacked over the four communities). The numerically correct average rates are detailed in Table 1. Comparisons across the three phases are set out in Table 2.

**Table 2:** Rates (per 1000 population) for trauma and assault compared across three phases

		2000–08	2009–12	2013–15	2000–08	2009–12	2013–15
		Phase 1	Phase 2	Phase 3	Phase 1	Phase 2	Phase 3
RFDS retrievals for trauma							
Community	1	21.5	14.7	17.5	BASE	−31.7%	−18.7%
	2	15.0	13.3	21.7	BASE	−11.7%	44.0%
	3	24.2	13.2	18.4	BASE	−45.3%	−24.0%
	4	21.0	16.5	12.8	BASE	−21.4%	−39.0%
	Average	20.5	14.4	17.6	BASE	−29.4%	−13.9%
Occurrences of assault recorded by QPS							
Community	1	123.3	74.5	118.6	BASE	−39.6%	−3.9%
	2	131.0	70.0	135.9	BASE	−46.6%	3.7%
	3	79.0	76.0	44.7	BASE	−3.9%	−43.4%
	4	102.4	66.9	71.3	BASE	−34.7%	−30.4%
	Average	108.9	71.8	92.6	BASE	−34.1%	−15.0%
Victims of assault recorded by QPS							
Community	1	73.2	54.9	83.9	BASE	−25.1%	14.5%
	2	84.4	55.1	83.6	BASE	−34.7%	−0.9%
	3	51.5	60.1	31.9	BASE	16.6%	−38.1%
	4	71.2	51.3	43.4	BASE	−28.0%	−39.1%
	Average	70.1	55.3	60.7	BASE	−21.1%	−13.4%

**Fig. 2:**
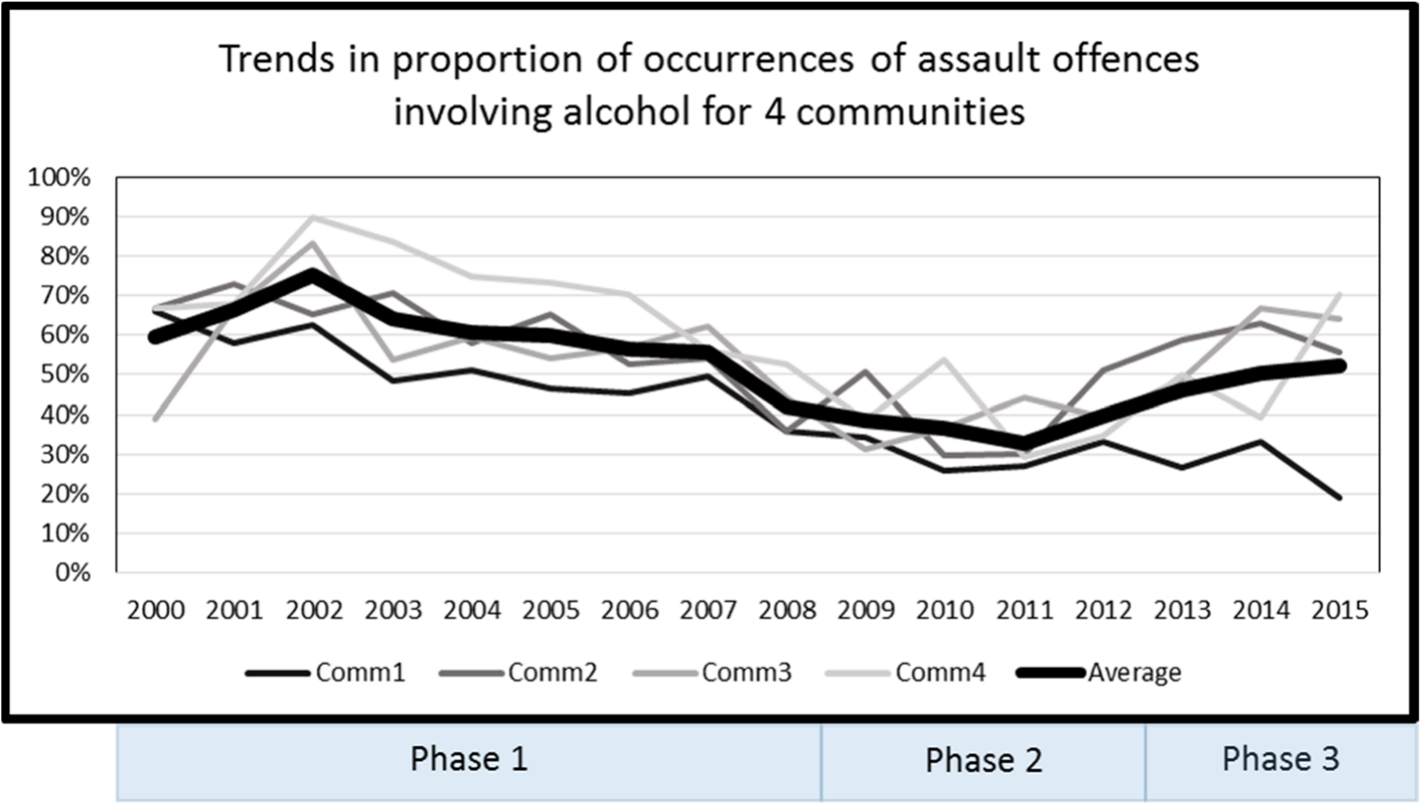
Trends in proportions of assault occurrences involving alcohol across three phases of restrictions

Time trends in the proportion of assault occurrences where alcohol was involved, including the average for all four communities, were depicted graphically (Fig. 2).

Since the study exclusively deals with population data (as opposed to samples), measures of statistical uncertainty (such as confidence intervals or statistical tests) are unnecessary and would be factually misplaced.

### Approvals

The Human Research Ethics Committees of James Cook University and the Cairns and Hinterland Health Services District provided relevant ethical approvals for the study to be conducted in these communities [52]. The QPS and RFDS Research Committees provided approvals for the study to be conducted and for data to be provided. We thank the Royal Flying Doctor Service (Queensland Section) and the Queensland Police Service for providing access to their data. The views expressed in this material are those of the author(s) and are not those of the Queensland Police Service or the Royal Flying Doctor Service. Responsibility for any errors of omission or commission remains with the author(s). The Queensland Police Service expressly disclaims any liability for any damage resulting from the use of the material contained in this publication and will not be responsible for any loss, howsoever arising, from use of or reliance on this material.

## Results

A total of 1019 aeromedical trauma retrievals were extracted from the RFDS database and used in the analysis for the four communities for 2000 to 2015 inclusive. During the same period, data for 5641 unique assault occurrences were provided by QPS. In 2936 (52%) of these assault occurrences, alcohol was recorded as being involved. During the observation period 2741 individuals were victims of an assault, as recorded by QPS. The resulting rates for retrievals and assaults for the four communities as well as the average over all communities over the single years of the observation period are numerically detailed in Table 1. Fig. 1a (retrievals), 1b (assault occurrences) and 1c (assault victims) display community specific time trends for the observed rates in Table 1.

Consistent with previous results [7, 10], Table 1 and Figs. 1a, b and c for the time trends show a decrease during Phase 1 at, or shortly after, the first round of restrictions commenced in 2002–203 and then a rise. Assault occurrences and assault victims, however, began to decline around two years sooner than retrievals towards the end of Phase 1 between 2005 and 2008, near the time ‘minimising harm’ provisions restricted alcohol supply from ‘catchment’ licensed premises in 2007 [19]. During the ‘dry as possible’ Phase 2 (2009–2012) all rates reached their lowest absolute and average levels. However, during Phase 3, from the beginning of the current review period after 2012, all rates showed a rising overall trend on average, approaching or exceeding pre-2008 levels. Of particular note is that assault occurrences rose substantially in communities 1, 2 and 4 in 2015, reaching pre-2008 levels. Trends in rates of assault victims recorded by police rose sharply in communities 1, 2 and 4 in 2015, also approaching or exceeding pre-2008 levels.

Figure 2 shows that trends in the proportion of assault occurrences where alcohol was involved began to decline from the very high levels (75% in 2002) seen towards the end of Phase 1; again before retrievals began to decline, following the trends in assault occurrences and assault victims. During the ‘dry as possible Phase 2, less than 40% of assault occurrences involved alcohol, also an historically low level. In Phase 3, however, communities 2, 3 and 4 showed a sharp rise to reach or exceed pre-2008 levels, while community 1 showed a steep decline (Fig. 2).

Table 2 displays community-specific and average rates (over the four communities) for retrieval rates for the nine years during: Phase 1 - from 2000 to 2008; Phase 2 - the four-year period (2009–2012) directly after the second round of restrictions; and Phase 3 - the last three years of available data (2013–2015). These rates were 20.5/1000; 14.4/1000 and 17.6/1000, respectively. Similarly, Table 2 indicates that average rates for QPS registered assault occurrences in each phase were 108.9/1000; 71.8/1000; and 92.6/1000 while rates for assault victims were 70.1/1000; 55.3/1000; and 60.7/1000, respectively. Taking Phase 1 average rates as the baseline, we see a substantial decrease in average retrieval rates by − 29.4% after the second round of restrictions but with a reduction by a lesser amount, − 13.9%, for the latest time period, Phase 3. The observed average decreases for assault occurrence rates are − 34.1% and − 15.0%, showing a reduction by a lesser amount compared with Phase 1. For assault victim rates, reductions compared with Phase 1 were − 21.1% in Phase2 and − 13.4% in Phase 3, indicating a slowing in the reduction of this indicator.

Table 2 also indicates that in Phase 3, only community 4 showed a continued average reduction in retrievals (− 39.0%), assault occurrences (− 30.4%) and assault victims (− 39.1%) relative to Phase 1. Community 3 saw a continued reduction in assault occurrences (− 43.4%) but did not see a reduction in assault victims until Phase 3 (− 38.1%) with a continued but slowing average reduction in retrievals (− 24.0%).

Communities 1 and 2 have not fared so well (Table 2). For retrievals, the average decline compared with Phase 1 has slowed in community 1 (− 18.7%) and dramatically reversed in community 2 (+ 44%). For assault occurrences, a continued average reduction since Phase 1 in community 1 is no longer apparent (− 3.9%) and a reduction may have reversed in community 2 (+ 3.7%). The average reduction in assault victims compared with Phase 1 has reversed in community 1 (+ 14.5%) and is no longer apparent in community 2 (− 0.9%).

## Discussion

In four remote Indigenous communities in far north Queensland, after initial reductions to historically low levels in 2010–2012, rates of indicators of violence and serious injury appear to have risen recently in most localities. Importantly, these rates are trending towards or have exceeded pre-2008 levels, particularly for aeromedical retrievals for serious injury in three communities and for assault occurrences and victims in two communities. Additionally, the average proportion of assault occurrences where QPS have recorded alcohol involved has generally returned to pre-2008 levels. By contrast, QPS estimated rates of violence against the person for Queensland as a whole for the period 2001–02 to 2014–15 showed a steadily decreasing trend [53]. As communities seem to have responded differently to the AMP policy in recent times, the substantial and socially-significant reductions in these important indicators achieved during Phase 2, the ‘dry as possible’ phase of Queensland’s AMP policy, have not been sustained in all communities to the same degree.

Evidence specific to the study communities has consistently pointed to the reduction of alcohol abuse being strongly correlated with a reduction in trauma and assault [7, 10, 32]. This association appears very similar for rates of assault occurrences (− 15.0%), assault victims (− 13.4%) and trauma retrievals (− 13.9%). This is largely due to the rise in assault victim rates in community 1 (+ 14.5%) and retrievals in community 2 (+ 44.0%) during Phase 3. On average, all communities taken together reached their greatest level of improvement in assault and retrieval rates during Phase 2, but with these important improvements becoming eroded in some localities in Phase 3. This is reinforced by the evidence for the rising trend in the proportion of assault occurrences with alcohol involved, which returned to pre-2008 levels in three of the four communities.

The data sets have probably reflected somewhat similar social pathologies and risks operating over the 16 years of observation, and so the cross-correlation of the time trends in rates, as depicted in Figs. 1a, b and c, is not unexpected. However, a generally strong correlation between the data sets over this wide spectrum of surrogates of person-to-person violence and over a long period of time gives us assurance that alcohol controls in these communities have had their intended effects, but only until the current Phase 3, when the time trends have begun to rise and also diverge.

### Limitations and strengths

Although interpersonal violence is intentional by definition, police records capture only that which is observed, detected or reported so that actual levels of violence may be under-reported and rates under-estimated. At the same time, observation, detection and reporting will increase where policing efforts are intensified, as happens from time to time in these communities when community disruption occurs. However, any reporting would be generally consistent across the period since policing levels in the four communities remained generally steady, notwithstanding strategic responses to urgent situations.

Severe traumas requiring aeromedical retrieval, on the other hand, are consistently likely to be prominent events in these small communities, often due to accidents as well as intentional person-to-person violence or dangerous acts. Any aeromedical retrieval due to interpersonal violence may well involve grievous or serious assault or a dangerous act with significant implications for serious injury in victims. Assaults in our data included ‘common assault’ as well as other acts and behaviours that would usually have lesser implications for serious physical injury among victims.

A weakness of the RFDS retrieval data is that it did not permit us to distinguish retrieval episodes for individuals while a strength of the QPS data is that it permitted unique occurrences and victims to be distinguished from multiple occurrences involving the same individuals. On balance, however, it is reasonable to conclude that the different data sets derived from these very authoritative sources, consistently depict a wide spectrum of person-to-person violence occurring in these communities and recorded in a consistent manner over time. In terms of assessing ‘safety’ in these communities in response to alcohol restrictions, the selected data sets are thus ideal; covering a uniquely wide range of violence measures.

### There was no explicit policy to relax restrictions in phase 3

Unlike Phase 1, where the first round of legislative reforms saw the introduction of carriage limits and minimising harm conditions on liquor retailers in ‘catchment’ licensed premises, and unlike Phase 2, where prohibition was implemented in three of the communities and restrictions tightened in the fourth from 2009, Phase 3 contained no direct legal intervention relaxing the restrictions. The recent changes observed in Phase 3 suggest the possibility that new social and contextual factors are beginning to have an influence beyond the direct effects of Queensland Government policy.

In theory, policies generally have a tendency to become exhausted [54, 55] and to ‘drift’ [56, 57] when the normal workings of government institutions undermine policy pre-conditions and legislative intent and where mobilisation of resources for policy implementation becomes weakened. In 2013, Queensland’s Department of Aboriginal and Torres Strait Islander and Multicultural Affairs commissioned a report by the Government Statistician to review the effects of s 168B of Queensland’s *Liquor Act 1992* which was the key legislative tool to enforce alcohol controls in AMP communities [58]. The data in the report indicate that since s 168B was enacted in 2002, more than 60% of the adult Indigenous residents of AMP communities in Queensland have been convicted of at least one s 168B breach [22]. There is no evidence to suggest that this shocking result was the intent of the Queensland legislature or bureaucracy in 2002. However, there is strong evidence that this exclusive criminalisation of some Indigenous community residents has increasingly undermined the implementation of AMPs and has threatened to exhaust community support for them [22, 24, 25].

In the background, during Phase 2, significant legal challenges to the legitimacy of AMPs were commenced [35, 36], one of which proceeded to an unsuccessful appeal in Australia’s High Court [36–38]. In May 2008, an Indigenous resident of Palm Island, one of Queensland’s AMP communities, was convicted of breaching s 168B by possessing other than the prescribed quantity of alcohol set down in Sched 1R of the *Liquor Regulation 2002* (Qld). This case required attention to international human rights conventions and the Australian Constitution [37, 38]. An appeal to her District Court conviction was rejected [34]. The grounds of appeal were that regulations made under Queensland’s *Liquor Act 1992* were invalid by reason of inconsistency with s 10 of Australia’s *Racial Discrimination Act 1975* (Cth). Section 10 of this Commonwealth act provides that a State law cannot have the effect that persons of a particular race do not enjoy a right enjoyed by other races, or do not enjoy it to the same degree. In dismissing the appeal, the Court of Appeal held that s 10 did not apply, as it was a ‘special measure’ taken for the sole purpose of securing the adequate advancement of the Indigenous residents of Palm Island and to prevent human rights violations, such as violation of the right of vulnerable groups to live in a safe environment [36].

The issue of consultation for ‘special measures’ and the proportionality aspects of AMPs in Australia’s Northern Territory have recently raised concerns for the Australian Parliamentary Joint Committee on Human Rights [59]. In its 2016 legislated review, the Committee noted that AMPs are measures which are based on race or ethnic origin within the meaning of the relevant human rights treaties [60, 61]. The Committee did not consider that AMPs were appropriately classified as ‘special measures’ but may be justified if shown to be a reasonable and proportionate measure rationally connected to the achievement of the purpose of the measure, with appropriate consultation with affected communities, and not to be held in place when that purpose is achieved [59]. On the evidence presented here, Queensland’s AMPs clearly achieved their purpose in the study communities in Phase 2 from 2009 to 2012. Given the rising trends in violence and injury in some of the communities seen in Phase 3, close continued monitoring and community consultation will be required to ensure that AMPs remain a reasonable and proportionate measure. The hard won successes in this difficult field must be retained.

## Conclusions

It is of great concern that rates of retrievals for serious injury, assault occurrences and victims show a generally increasing trend since 2012 in these four study communities. This is in the absence of any substantial policy change affecting alcohol’s legal availability. The underlying causes for these trends warrant urgent, refreshed policy action informed by rigorous objective monitoring to ensure the successes gained by AMPs in Queensland are not placed in further jeopardy.

## Abbreviations

AMP: Alcohol management plan
LGC: Local Government Council
QPS: Queensland Police Service
RFDS: Royal Flying Doctor Service

## References

[1] World Health Organization: Global status report on alcohol and health*.* 2014, Geneva: world health organization. Available from: http://apps.who.int/iris/bitstream/10665/112736/1/9789240692763_eng.pdf?ua=1 Accessed 8 March 2018.

[2] Brand DA, Saisana M, Rynn LA, Pennoni F, Lowenfels AB (2007). Comparative analysis of alcohol control policies in 30 countries. PLoS Med.

[3] Davison CM, Ford CS, Peters PA, Hawe P (2011). Community-driven alcohol policy in Canada's northern territories 1970-2008. Health Policy.

[4] Kovas AE, McFarland BH, Landen MG, Lopez AL, May PA (2008). Survey of American Indian alcohol statutes, 1975-2006: evolving needs and future opportunities for tribal health. J Stud Alcohol Drugs.

[5] d'Abbs P (2015). Widening the gap: the gulf between policy rhetoric and implementation reality in addressing alcohol problems among indigenous Australians. Drug Alcohol Rev..

[6] Chiu AY, Perez PE, Parker RN (1997). Impact of banning alcohol on outpatient visits in Barrow, Alaska. JAMA.

[7] Margolis SA, Ypinazar VA, Muller R (2008). The impact of supply reduction through alcohol management plans on serious injury in remote indigenous communities in remote Australia: a ten-year analysis using data from the Royal Flying Doctor Service. Alcohol Alcohol.

[8] Berman M, Hull T, May P (2000). Alcohol control and injury death in Alaska native communities: wet, damp and dry under Alaska's local option law. J Stud Alcohol.

[9] Douglas M (1998). Restriction of the hours of sale of alcohol in a small community: a beneficial impact. A N Z J Public Health.

[10] Margolis SA, Ypinazar VA, Muller R, Clough A (2011). Increasing alcohol restrictions and rates of serious injury in four remote Australian indigenous communities. Med J Aust.

[11] Muhunthan J, Angell B, Hackett ML, Wilson A, Latimer J, Eades AM (2017). Global systematic review of indigenous community-led legal interventions to control alcohol. BMJ Open.

[12] Queensland Department of the Premier and Cabinet: Meeting Challenges, Making Choices: the Queensland Government's Response to the Cape York Justice Study. 2002, Brisbane, Queensland: Queensland Government available from: http://pandora.nla.gov.au/pan/24611/20020516-0000/www.premiers.qld.gov.au/about/community/pdf/justice_study_response.pdf accessed: 8 March 2018.

[13] Fitzgerald T. Cape York Justice Study. 2001, Brisbane: Queensland Department of the Premier and Cabinet. Available from: http://www5.austlii.edu.au/au/journals/AUIndigLawRpr/2001/56.html. Accessed 21 July 2017 accessed: 8 March 2018.

[14] Gray D, Saggers S, Atkinson D, Sputore B, Bourbon D (2000). Beating the grog: an evaluation of the Tennant Creek liquor licensing restrictions. Aust N Z J Public Health.

[15] Commonwealth of Australia: 2016 Review of Stronger Futures measures. 2016, Canberra: Parliamentary Joint Committee on Human Rights. Available from: https://www.aph.gov.au/Parliamentary_Business/Committees/Joint/Human_Rights/Committee_Inquiries/strongerfutures2/Final_report Accessed: 8 March 2018.

[16] State of Queensland. The Aboriginal and Torres Strait Islander Women’s Task Force on Violence Report. Department of Aboriginal and Torres Strait Islander Policy and Development. Brisbane: 1999. Available from: http://www.indigenouschamber.org.au/wp-content/uploads/2017/03/Aboriginal-Torres-Strait-Islanders-Womens-Task-Force-on-Violence-Report.pdf. Accessed 8 Mar 2018.

[17] Queensland Parliament. Liquor Act 1992. Available from: https://www.legislation.qld.gov.au/view/pdf/2014-06-20/sl-2002-0212. Accessed 8 March 2018.

[18] Queensland Parliament. Liquor Regulation (2002). Available from: https://www.legislation.qld.gov.au/view/pdf/2017-08-25/sl-2002-0212 Accessed 8 March 2018.

[19] Clough AR, Bird K (2015). The implementation and development of complex alcohol control policies in indigenous communities in Queensland (Australia). Int J Drug Policy..

[20] Queensland Parliament. Aboriginal and Torres Strait Islander Communities (Justice, Land and Other Matters) and Other Acts Amendment Act 2008, Stat. 30 (2008). Available from: https://www.legislation.qld.gov.au/view/html/asmade/act-2008-030#act-2008-030 Accessed 8 March 2018.

[21] Queensland Parliament. Aboriginal and Torres Strait Islander Communities (Justice, Land and Other Matters) and Other Acts Amendment Bill 2008, 52 Sess. (2008). Available from: https://www.legislation.qld.gov.au/view/html/bill.first/bill-2008-1344. Accessed 8 Mar 2018.

[22] Clough AR, Margolis SA, Miller A, Shakeshaft A, Doran CM, McDermott R (2017). Alcohol management plans in aboriginal and Torres Strait islander (indigenous) Australian communities in Queensland: community residents have experienced favourable impacts but also suffered unfavourable ones. BMC Public Health.

[23] Usher K, Woods C, Lynch P, Pointing SB, Budden L, Barker R (2017). Is population flow an unintended consequence of alcohol management plans?. J Clin Nurs.

[24] Robertson JA, Fitts MS, Clough AR (2017). Unintended impacts of alcohol restrictions on alcohol and other drug use in indigenous communities in Queensland (Australia). Int J Drug Policy..

[25] Clough AR, Margolis SA, Miller A, Shakeshaft A, Doran CM, McDermott R (2016). Alcohol control policies in indigenous communities: a qualitative study of the perceptions of their effectiveness among service providers, stakeholders and community leaders in Queensland (Australia). Int J Drug Policy.

[26] Fitts MS, Robertson J, Towle S, Doran CM, McDermott R, Miller A (2017). 'Sly grog' and 'homebrew': a qualitative examination of illicit alcohol and some of its impacts on indigenous communities with alcohol restrictions in regional and remote Queensland (Australia). BMC Res Notes.

[27] Landen MG, Beller M, Funk E, Propst M, Middaugh J, Moolenaar RL (1997). Alcohol-related injury death and alcohol availability in remote Alaska. JAMA.

[28] Schechter EJ (1986). Alcohol rationing and control systems in Greenland. Contempt Drug Probl.

[29] Hogan E, Boffa J, Rosewarne C, Bell S, Chee DA (2006). What price do we pay to prevent alcohol-related harms in aboriginal communities? The Alice Springs trial of liquor licensing restrictions. Drug Alcohol Rev.

[30] d'Abbs PH (1998). Out of sight, out of mind? Licensed clubs in remote aboriginal communities. A N Z J Public Health..

[31] Gladman DJ, Hunter EMM, McDermott RA, Merritt TD, Tulip FJ (1997). Study of injury in five Cape York communities. Cairns: Australian Institute of Health and Welfare, National Injury Surveillance Unit. Queensland health.

[32] Editors. Cape York Justice Study - Digest. Aust Indig Law Rep. 2001;56 Available from: http://www5.austlii.edu.au/au/journals/AUIndigLawRpr/2001/56.html Accessed 8 March 2018.

[33] Pearson N (2001). Cape York partnerships and Apunipima Cape York health council. Outline of a grog and drugs (and therefore violence) strategy.

[34] R v Maloney. Queensland Court of Appeal; [2012] 262 FLR 172.

[35] Aurukun Shire Council v Chief Executive, Office of Liquor Gaming and Racing in the Department of Treasury*.* Queensland Court of Appeal; [2010] QCA 37.

[36] Joan Monica Maloney v The Queen*.* High Court of Australia; [2013] HCA 28.

[37] Rice S (2013). Joan Monica Maloney v the Queen [2013] HCA 28. Indigenous Law Bull.

[38] Wall P (2014). The high court of Australia's approach to the interpretation of international law and its use of international legal materials in Joan Monica Maloney v the Queen [2013] HCA 28. Melbourne J Int Law.

[39] Department of the Prime Minister and Cabinet: Review of the Stronger Futures in the Northern Territory Act *(*2012*).* 2016, Canberra: commonwealth of Australia. Available from: https://www.aph.gov.au/Parliamentary_Business/Committees/Joint/Human_Rights/Committee_Inquiries/strongerfutures2/Final_report Accessed 8 March 2018.

[40] Australian Parliamentary Joint Committee on Human Rights. Stronger Futures in the Northern Territory Act 2012 and related legislation: Measures to address alcohol abuse [2013] AUPJCHR 324 (26 June 2013). Available from: http://www.austlii.edu.au/cgi-bin/sinodisp/au/other/AUPJCHR/2013/324.html?stem=0&synonyms=0&query=stronger%20futures. Accessed 8 March 2018.

[41] Krug E, Dahlberg L, Mercy J, Zwi A, Lozano R. 2002, World Report on Violence and Health. Geneva: World Health Organization. Available from: http://www.who.int/violence_injury_prevention/violence/world_report/en/ accessed 8 Mar 2018.

[42] Graham K. Social drinking and aggression. In: Mattson M, editor. Neurobiology of aggression: understanding and preventing violence, 1st ed. Totowa, New Jersey: Humana Press; 2003.

[43] Taylor B, Irving HM, Kanteres F, Room R, Borges G, Cherpitel C (2010). The more you drink, the harder you fall: a systematic review and meta-analysis of how acute alcohol consumption and injury or collision risk increase together. Drug Alcohol Depend.

[44] Cherpitel CJ, Ye Y, Bond J, Borges G, Monteiro M, Chou P (2015). Alcohol attributable fraction for injury morbidity from the dose-response relationship of acute alcohol consumption: emergency department data from 18 countries. Addiction.

[45] Giancola PR (2015). Development and evaluation of theories of alcohol-related violence: covering a 40-year span. Subst Use Misuse.

[46] Queensland Government Statistician's Office. Australian Standard Offence Classification (Queensland Extension)*.* 2008, Brisbane, Australia: Queensland government. Available from: http://www.qgso.qld.gov.au/about-statistics/statistical-standards/queensland/qasoc-2008.pdf Accessed 8 March 2018.

[47] Talpins SK, Robertson R, Holmes, Dunagan M. Alcohol and crime. In Boyle P, Boffetta P, Lowenfels AB, Burns H, Brawley O, Zatonski W, Rehm J, editors. Alcohol: science, policy and public health. Oxford University Press; 2013. p. 202–8.

[48] World Health Organization: Interpersonal violence and alcohol policy briefing**.** 2018, Geneva: world health organization; 2018 Available from: http://www.who.int/violence_injury_prevention/violence/world_report/factsheets/pb_violencealcohol.pdf Accessed 8 Mar 2018.

[49] West C, Muller R, Clough AR (2017). Injuries and alcohol management plans in remote indigenous communities: a two-community comparison. Inj Prev.

[50] Queensland Government Statistician's Office: Population projections*.* 2018, Brisbane, Australia: Queensland Government Available from: http://www.qgso.qld.gov.au/subjects/demography/population-projections/ Accessed 8 Mar 2018.

[51] Australian Bureau of Statistics. Census Community Profiles*.* 2016, Canberra, Australia: commonwealth of Australia. Available from: http://www.censusdata.abs.gov.au/census_services/getproduct/census/2016/communityprofile/036?opendocument Accessed 8 March 2018.

[52] Clough AR, Fitts MS, Robertson JA, Shakeshaft A, Miller A, Doran CM (2014). Study protocol--alcohol management plans (AMPs) in remote indigenous communities in Queensland: their impacts on injury, violence, health and social indicators and their cost-effectiveness. BMC Public Health.

[53] Queensland Police Service: Annual statistical review 2015/16, Brisbane, Australia: Queensland Government Available from: https://www.police.qld.gov.au/corporatedocs/reportsPublications/statisticalReview/Documents/2015-16/AnnualStatisticalReview_2015-16.pdf Accessed 10 July 2018.

[54] Cerna L. The Nature of Policy Change and Implementation: A Review of Different Theoretical Approaches. Paris: Organisation for Economic Co-operation and Development (OECD); 2013. Available from: file:///C:/Users/jc166639/Documents/amp/PAPERS/Med_J_Aust/The%20Nature%20of%20Policy%20Change%20and%20Implementation.pdf. Accessed 8 March 2018.

[55] Streeck W, Thelen K (2005). Beyond continuity : institutional change in advanced political economies.

[56] Kam C (2000). Not just parliamentary 'Cowboys and Indians': ministerial responsibility and bureaucratic drift. Governance.

[57] McCubbins MD, Noll RG, Weingast BR (1987). Administrative procedures as instruments of political control. J Law Econ Organ.

[58] Queensland Government Statistician's Office: Alcohol Management Plan Review: breach of alcohol restrictions in Indigenous communities and associated contact with the criminal justice system*,* 2013. Brisbane, Queensland: Queensland Government.

[59] Parliamentary Joint Committee on Human Rights: Parliamentary Joint Committee on Human Rights- Human rights scrutiny report*.* 2017, Canberra: Commonwealth of Australia. Available from: https://www.aph.gov.au/Parliamentary_Business/Committees/Joint/Human_Rights/Scrutiny_reports/2017/Report_4_of_2017. Accessed 8 March 2018.

[60] United Nations General AssemblyInternational Covenant on Economic, Social and Cultural Rights, 16 December 1966, United Nations, Treaty Series, vol. 993, p. 3. 1966,Vienna: United Nations. Available at: http://www.refworld.org/docid/3ae6b36c0.html Accessed 20 April 2017.

[61] United Nations Human Rights Office of the High Commissioner. International Convention on the Elimination of All Forms of Racial Discrimination 1965. Available from: http://www.ohchr.org/EN/ProfessionalInterest/Pages/CERD.aspx. Accessed 8 March 2018.

